# Nerve Growth Factor in Pediatric Brain Injury: From Bench to Bedside

**DOI:** 10.3390/ph18060929

**Published:** 2025-06-19

**Authors:** Lorenzo Di Sarno, Serena Ferretti, Lavinia Capossela, Antonio Gatto, Valeria Pansini, Anya Caroselli, Luigi Manni, Marzia Soligo, Antonio Chiaretti

**Affiliations:** 1 Department of Pediatrics, Fondazione Policlinico Universitario “A. Gemelli”, IRCCS, 00168 Rome, Italy; lorenzo.disarno1@guest.policlinicogemelli.it (L.D.S.); lavinia.capossela1@guest.policlinicogemelli.it (L.C.); antonio.gatto@policlinicogemelli.it (A.G.); valeria.pansini@policlinicogemelli.it (V.P.); 2 Department of Pediatrics, Fondazione Policlinico Universitario “A. Gemelli”, IRCCS, Università Cattolica del Sacro Cuore, 00168 Rome, Italy; anya.caroselli01@icatt.it (A.C.); antonio.chiaretti@policlinicogemelli.it (A.C.); 3 Istituto di Farmacologia Traslazionale, Consiglio Nazionale delle Ricerche (CNR), 00133 Rome, Italy; luigi.manni@ift.cnr.it (L.M.); marzia.soligo@ift.cnr.it (M.S.)

**Keywords:** nerve growth factor, brain injury, children, NGF, neuroprotection

## Abstract

**Background:** Traumatic brain injury (TBI) and hypoxic–ischemic encephalopathy (HIE) are major causes of long-term neurological disability in children, with limited options for effective neuronal recovery. Recent research has highlighted the therapeutic potential of nerve growth factor (NGF) in promoting neural repair through mechanisms such as neuroprotection, neurogenesis, and the modulation of neuroinflammation. This review evaluates the current evidence on NGF as a treatment strategy for pediatric brain injury, emphasizing its mechanisms of action and translational clinical applications. **Methods:** A comprehensive review was conducted using the PubMed, Scopus, and Cochrane CENTRAL databases to identify studies published between 1 January 1978 and 1 March 2025, investigating NGF in the context of brain injury. The inclusion criteria comprised studies assessing neurological outcomes through clinical scales, biochemical markers, neuroimaging, or electrophysiological examinations. **Results:** Seventeen studies met the inclusion criteria, encompassing both preclinical and clinical research. Preclinical models consistently demonstrated that NGF administration reduces neuroinflammation, enhances neurogenesis, and supports neuronal survival following TBI and HIE. Clinical studies, including case reports of pediatric patients treated with intranasal NGF, reported improvements in motor and cognitive function, neuroimaging findings, and electrophysiological parameters, with no significant adverse effects observed. **Conclusions:** NGF demonstrates significant promise as a neuroprotective and neuroregenerative agent in pediatric brain injury, with both experimental and early clinical evidence supporting its safety and efficacy. Large-scale controlled clinical trials are warranted to validate these preliminary findings and to determine the optimal dosage regimens and administration schedules for NGF in the treatment of TBI and HIE.

## 1. Introduction

Traumatic brain injury (TBI) remains a major public health concern, particularly among pediatric and young adult populations, as it is a leading cause of disability and mortality [1]. The complex pathophysiology of TBI involves an initial mechanical injury to brain tissue, followed by a cascade of molecular events that contribute to neuronal damage and dysfunction [2]. These include inflammation, excitotoxicity, oxidative stress, mitochondrial dysfunction, and the disruption of the blood–brain barrier, which together impair neural repair and regeneration [3,4,5]. As a result, TBI often leads to long-term cognitive, behavioral, and motor deficits, significantly affecting the quality of life of affected individuals [6]. At present, there is limited evidence on effective treatments for neuronal recovery after TBI. Similarly, hypoxic–ischaemic encephalopathy (HIE), a critical neonatal brain injury caused by oxygen deprivation and impaired cerebral blood flow, remains a condition with limited treatment options and frequent long-term neurological deficits [7]. Currently, therapeutic hypothermia stands as the only clinically validated treatment, reducing mortality and disability when started within six hours of birth. However, its efficacy is partial, particularly in severe HIE cases, and the outcomes may still include cognitive impairments, motor dysfunction, or epilepsy. While emerging therapies such as erythropoietin, stem cell transplantation, and melatonin show preclinical promise as neuroprotectants or regenerative agents, none have yet achieved standard clinical adoption [8,9].

In recent years, growing interest has focused on the potential therapeutic benefits of neurotrophic factors, specifically nerve growth factor (NGF), in promoting recovery following brain injury [10,11]. NGF, a key protein involved in the growth, maintenance, and survival of neurons, has shown promise in mitigating the effects of brain injury by promoting neuroprotection, neurogenesis, and neuroplasticity [12]. Studies suggest that NGF may reduce neuroinflammation, prevent neuronal loss, and support the regeneration of damaged neural circuits, thus offering hope for improved outcomes in brain-injured patients [13,14,15]. A significant challenge in the therapeutic delivery of NGF lies in identifying the most effective route of administration to directly target the central nervous system (CNS). Among the various methods available, intraventricular administration has been employed due to its ability to bypass the blood–brain barrier and deliver agents directly into the cerebrospinal fluid. However, this approach is highly invasive, involving surgical intervention, which limits its widespread clinical application and raises concerns regarding patient safety and procedural complications. In contrast, the intranasal route of administration has emerged as a promising non-invasive alternative. This method exploits the unique anatomical and physiological characteristics of the olfactory pathway, allowing therapeutic molecules to bypass the blood–brain barrier and reach the CNS efficiently. Empirical evidence supports the efficacy of intranasal delivery in transporting adequate concentrations of NGF directly to the brain via the olfactory nerve, thereby enhancing the potential for clinical translation [13].

This review aims to explore the current body of literature on the role of NGF in brain injury, encompassing both traumatic and hypoxic–ischemic etiologies, highlighting its mechanisms of action, potential therapeutic applications, and the challenges in translating these findings into clinical practice. By synthesizing the latest research, this review seeks to provide a comprehensive understanding of NGF’s role in brain injury recovery and its potential as a therapeutic agent for enhancing brain repair and functional recovery.

## 2. Materials and Methods

A literature search was conducted to identify NGF-related studies and reports published between 1 January 1978 and 1 March 2025; the following electronic databases were systematically searched: PubMed; Scopus; and Cochrane Central Register of Controlled Trials (CENTRAL). The search was guided by the Preferred Reporting Items for Systematic Reviews and Meta-Analysis (PRISMA) method [16].

The research strings were as follows:

- Nerve growth factor;
- NGF;
- Neurotrophins;
- Neuroplasticity;
- Traumatic brain injury;
- TBI;
- HIE;
- Hypoxic–Ischemic Encephalopathy;
- Brain injury;
- NGF AND children.

To be considered eligible for the review, papers had to include the following components: (1) children or preclinical models with diagnosis of brain injury; (2) children or preclinical models who also received NGF as treatment; (3) neurological clinical outcomes evaluated by assessment scales—Glasgow Coma Scale (GCS), Coma recovery scale-revised (CRS-R), etc.—by radiological or biochemical examinations, or by electroencephalography (EEG). We excluded: non-English language papers and studies in which neurological outcomes were not expressed in assessment scales or evaluated by radiological examinations or EEG. We did not include papers published in languages other than English. We acknowledge that this represents a significant limitation of our study. However, it is important to note that this restriction is consistent with the methodology employed by the vast majority of research in the field of neurotrophins. Consequently, it aligns with common practice and standards within this area of investigation.

The current investigation mainly concentrated on randomized placebo–control studies, case–control studies, retrospective and prospective observational studies, systematic reviews, and meta-analyses.

The article selection method was supported independently by three reviewers (LC, SF, and LDS). All significant articles discovered were further scrutinized for extra references that did not appear in the preliminary examination.

Review or commentary papers without original data were eliminated, whereas their contents were used to clarify collected information.

The quality of the trials was thoroughly evaluated, and the following potential biases have been assessed: random classification group (selection bias); similarity of patients at baseline concerning the most significant prognostic indicators (homogeneity bias); allocation hiding (selection bias); blinding of outcome valuation (detection bias); partial outcome data (attrition bias); evading of co-interventions (co-intervention bias); and report of drop out (drop out bias). Case series and preclinical studies are inherently prone to bias due to their uncontrolled design, lack of randomization, and potential selection and information biases, making them unreliable for definitive efficacy assessment. However, they play a crucial role in generating hypotheses by revealing novel observations and potential treatment effects that guide further research.

## 3. Results

**Figure 1:**
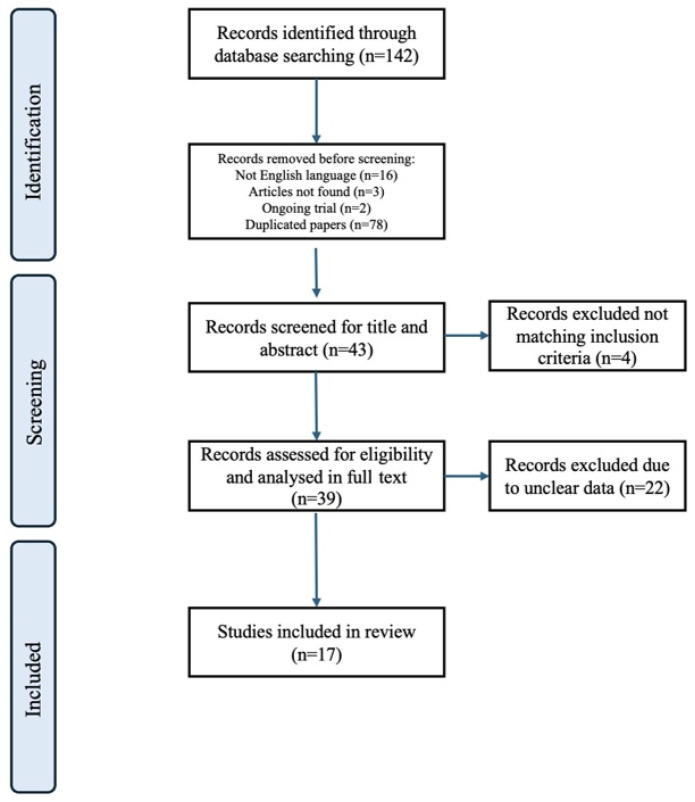
Flow diagram of literature selection.

Overall, we identified 142 records through database searching. As a first step, we excluded 16 articles in a non-English language, 3 records whose related articles were not available, 2 articles concerning ongoing trials, and 78 duplicated papers. As a second step, we eliminated 4 records by evaluating only title and abstract because they did not match the inclusion criteria we mentioned before. Of the remaining 39 studies, we excluded 22 through a further discussion among the authors on the reliability of the data (15 because of poorly reported data, 5 because of outcomes not matching inclusion criteria upon full-text review, and 2 because of participant dropout due to ineffective treatment or adverse events). Thus, 17 selected articles were included in the review. The detailed selection of the literature is shown in Figure 1. The characteristics of all included studies are summarized in Table 1 and Table 2**.**

### 3.1. Nerve Growth Factor in Traumatic Brain Injury

Severe TBI is the leading cause of death and acquired disability in children and young adults in industrialized countries [13]. The secondary damage following a brain injury involves multiple mechanisms, including the activation of inflammatory pathways with the production of cytokines and free radicals, as well as an imbalance in the synthesis and activity of neurotrophins. This often results in cell loss and the impairment of cholinergic functions. In recent years, new therapeutic strategies, both pharmacological and non-pharmacological, have been developed to limit and prevent secondary damage after severe TBI, but the results are still poor [9].

A prospective observational clinical study showed that the IL-6 and NGF levels in the cerebrospinal fluid (CSF) of children with severe TBI were higher than those in control subjects, indicating the involvement of inflammatory mediators in the brain’s response to injury [17]. Other reports have highlighted the critical role of NGF and doublecortin (DCX) in neurogenesis and neuronal repair [4,18]. Specifically, measuring the NGF and DCX levels in the CSF of children with TBI at two different time points (2 h and 48 h after the injury, referred to as T1 and T2), the authors revealed that the children with better outcomes had higher NGF levels than those with poorer outcomes. Furthermore, increased DCX expression was only observed in patients whose NGF levels rose from T1 to T2 [18].

NGF has also been shown to correlate significantly with injury severity and better neurological recovery [19].

However, interesting data has emerged regarding the therapeutic use of NGF after TBI. Summarizing the most pivotal experimental findings, one of the main benefits of NGF delivery in TBI therapy is the reduction in neuroinflammation [20]. NGF, known for its anti-inflammatory properties, regulates immune cell phenotypes and the production of inflammatory mediators. In the CNS, it modulates astrocyte and microglia metabolism and function, limiting astrogliosis and glial scar formation by arresting astrocyte cell cycle progression [13]. Intranasal NGF administration in TBI patients enhances brain energy metabolism, as shown by an increased Positron Emission Tomography/Computed Tomography (PET/CT) fluorodeoxyglucose uptake [13,14,21]. NGF may also regulate astrocyte–neuron metabolic interactions and compete with proNGF at astrocytic p75 neurotrophin receptor (p75NTR) receptors to promote neurorepair [20]. Additionally, NGF shifts microglia toward a neuroprotective phenotype via tropomyosin receptor kinase A (TrkA) activation, reducing pro-inflammatory cytokine production and inhibiting Nuclear Factor-κB (NF-κB), c-Jun NH 2-terminal kinase (JNK) pathways, and glycolysis [13,20].

NGF treatment after TBI can modulate protein metabolism, affecting amyloid-beta (Aβ), tau, and α-Synuclein (α-Syn) aggregation. By reducing pro-inflammatory cytokines, NGF promotes the microglial clearance of Aβ and lowers its cytotoxicity [20]. NGF also regulates amyloid precursor protein (APP) processing through TrkA binding, shifting its metabolism toward a non-amyloidogenic pathway [13]. Furthermore, NGF influences tau turnover, reducing hyperphosphorylation, while proNGF via p75NTR activation promotes tau pathology; thus, exogenous NGF may rebalance this system and provide neuroprotection [20]. After trauma, increased proNGF contributes to secondary brain damage, and blocking its effects has shown protective outcomes [13]. Regarding α-Syn, NGF upregulates its expression in vitro, suggesting a role in synaptic plasticity, although a direct link between NGF signaling and pathological α-Syn aggregation remains unproven [20].

NGF regulates mitochondrial function and can counteract mitochondrial dysfunction caused by TBI [13]. TrkA receptors in mitochondria protect against calcium-induced permeability transition, while NGF also boosts antioxidant defenses and reduces reactive oxygen species (ROS) production [20]. Additionally, NGF improves brain perfusion through its pro-angiogenic effects, promoting Vascular Endothelial Growth Factor (VEGF) production, endothelial cell proliferation, nitric oxide release, and vascular innervation [13]. Intranasal NGF stimulates neo-angiogenesis post stroke and enhances brain blood flow and metabolism, mechanisms relevant to its therapeutic benefits observed in TBI patients [20].

Analyzing interventional studies currently available in the literature, the first promising results emerged from experimental studies on rats [22,23,24,25]. In particular, preclinical studies on TBI models suggest that the intranasal delivery of NGF has the potential to modify disease progression, potentially preventing the onset of disabilities [25]. In these models, NGF is typically administered either immediately before or shortly after the injury. While four [22,23,24,25] out of five studies reported beneficial effects of intranasal NGF treatment, just one study [26] found no significant improvement in TBI-induced impairments. The evidence shows that intranasal NGF administration in TBI-exposed rats enhances locomotor performance and spatial memory, reduces Aβ accumulation [20], mitigates cerebral edema formation, lowers inflammatory cytokine production, and decreases apoptosis linked to mitochondrial dysfunction [22]. Furthermore, NGF treatment appears to inhibit glycogen synthase kinase-3β activity, thereby reducing tau hyperphosphorylation [23]. In the most recent preclinical study, Manni et al. investigated the impact of acute intranasal administration of NGF in a rat model of TBI complicated by hypoxia [25]. Human recombinant NGF (50 μg/kg) was administered intranasally immediately following the traumatic event, and motor behavior assessments, as well as morphometric and biochemical analyses, were performed 24 h later. Their findings demonstrated that NGF treatment effectively prevented the development of motor impairments typically induced by TBI. Moreover, the intervention significantly reduced reactive astrogliosis, microglial activation, and interleukin-1β (IL-1β) levels, pathological changes that were otherwise observed equally in both the lesion site and the hypothalamus following trauma [25].

Based on these encouraging results, this treatment has been tested in humans, particularly in children, as there are currently no effective therapeutic strategies for patients with severe TBI, but only palliative care options. Furthermore, the intranasal administration route proved to be an innovative and minimally invasive alternative that makes the treatment of patients in this age group safe and simple [21].

Notably, the first case of a pediatric patient treated with intranasal NGF 6 months after severe TBI was a 4-year-old boy who showed clinical improvement and positive changes in cerebral PET/CT, cerebral Single Photon Emission Computed Tomography (SPECT), cerebral Magnetic Resonance Imaging (MRI), electroencephalogram (EEG), and visual evoked potentials (VEP) after treatment. In fact, he demonstrated improvements in voluntary motor control, facial mimicry, phonation, attention, and verbal comprehension, as well as in the ability to cry, cough reflex, oral motor function, feeding ability, and bowel and bladder control [21].

In 2023, the treatment experience of three children suffering from post-traumatic unresponsive wakefulness syndrome who underwent intranasal human-recombinant NGF (hr-NGF) administration was published. The therapy, administered at least 6 months after the TBI, led to significant clinical improvements, including reduced spasticity and the recovery of facial mimicry, voluntary movements, oral motor function, verbal comprehension, attention, cough reflex, crying ability, and feeding abilities, which were reflected in enhanced neurological scores [14]. These clinical gains were supported by improved findings in cerebral functional imaging (PET/CT and SPECT) and electrophysiological evaluations (EEG and Power Spectral Density) [14].

Another study reported the case of a 14-year-old boy affected by a diffuse axonal injury consequent to severe TBI [27]. After intranasal hr-NGF administration, the authors observed a significant improvement of the patient’s radiologic functional pattern, evaluated with cerebral SPECT and brain PET/CT scans. In addition, an interesting enhancement was found also in cognitive functions: the boy showed an improvement in communication strategy planning, attention, memory, execution abilities, and verbal expression [27].

The same research group also described the case of a 4-year-old child victim of severe TBI, following a car accident. The child exhibited significant neurological impairment. Following hr-NGF administration, which was performed 9 months after the trauma, he achieved significant gains in motor function, attaining the ability to stand and ambulate independently. Neurocognitive evaluations revealed enhancements across multiple domains, notably in verbal comprehension and executive functions. EEG findings demonstrated a reduction in epileptiform discharges [15].

A pivotal finding is that no side effects were reported in any study, but further studies, especially with a larger sample size, may be useful in order to detect the safety and effects of NGF treatment. In addition, it would be worth evaluating whether the earlier administration of intranasal NGF in patients affected by severe TBI may have better effects than the already successful remote treatment, based on what has been shown in preclinical studies.

A general summary is represented in Table 1.

**Table 1:** Overview of current studies on NGF in TBI.

Study Type	Population	Intervention	Key Findings	Reference
Preclinical	90 adult rats	50 mg/day of NGF, delivered intranasally, for 14 days	NGF delivery promoted the decrease in TBI-induced Aβ deposits and improved TBI-induced functional impairment (*p* < 0.05)	Tian et al., 2012 [22]
Preclinical	24 adult rats	5 mg/day of NGF, delivered intranasally, for 12, 24, and 72 h	NGF treatment promoted the decrease in TBI-increased aquaporin-4 content and brain edema and the reduction in apoptosis by up-regulation of Bcl-2 and down-regulation of caspase-3 (*p* < 0.05)	Lv et al., 2013 [23]
Preclinical	132 adult rats	5 mg/day of NGF, delivered intranasally, for 3 days	NGF therapy promoted the attenuation of TBI-induced tau hyperphosphorylation and the decrease in IL-1β secretion (*p* < 0.05)	Lv et al., 2014 [24]
Preclinical	48 adult rats	5 mg/day of NGF, delivered intranasally, for 7 days	NGF treatment showed no effects on functional motor recovery after TBI (*p* < 0.05)	Young et al., 2015 [26]
Preclinical	136 adult rats	50 μg/kg of NGF, intranasally delivered in three rounds in a single day	NGF therapy prevented the onset of TBI-induced motor disabilities and reduced microglial activation, reactive astrogliosis, and IL-1β expression (*p* < 0.05)	Manni et al., 2023 [25]
Clinical	A 4-year-old boy	0.1 mg/kg of murine NGF, delivered intranasally, twice a day for 10 consecutive days, for 4 cycles, at 1 month distance each	NGF treatment improved voluntary motor control, facial mimicry, phonation, attention and verbal comprehension, ability to cry, cough reflex, oral motor function, feeding ability, and bowel and bladder control	Chiaretti et al., 2017 [21]
Clinical	3 children aged 3 to 10 years	50 µg/kg of hr-NGF, administered intranasally, three times a day for 7 consecutive days for 4 cycles, at 1 month distance each.	NGF therapy reduced spasticity and improved the recovery of facial mimicry, voluntary movements, oral motor function, verbal comprehension, attention, cough reflex, crying ability, and feeding abilities	Gatto et al., 2023 [14]
Clinical	A 14-year-old boy	50 µg/kg of hr-NGF, administered intranasally, three times a day for 7 consecutive days for 4 cycles, at 1 month distance each	NGF administration improved radiological functional assessment, cognitive processes, memory, communication strategy, execution skills, attention, and verbal expression	Capossela et al., 2024 [27]
Clinical	A 4-year-old boy	50 µg/kg of hr-NGF, administered intranasally, three times a day for 7 consecutive days for 4 cycles, at 1 month distance each	NGF therapy improved motor function, verbal comprehension, executive functions, and EEG pattern	Di Sarno et al., 2025 [15]

### 3.2. Nerve Growth Factor in Neonatal Hypoxic–Ischemic Brain Injury

NGF has garnered considerable recent scientific interest for its potential neuroprotective features in neonatal HIE [28]. This condition results from perinatal asphyxia, which causes insufficient oxygen and blood flow to the developing brain [29]. HIE is a major cause of neonatal mortality and permanent neurological sequelae, including cerebral palsy, cognitive impairment and epilepsy, affecting a large number of patients worldwide each year [30]. Current treatments, such as therapeutic hypothermia, focus on reducing the severity of the injury, but they do not always prevent long-term complications.

The pathophysiology of HIE involves a complex cascade of events, including primary energy failure, excitotoxicity, oxidative stress, inflammation, and apoptotic cell death, which collectively contribute to widespread neuronal and glial injury [31].

NGF exerts its biological effects primarily by binding to the high-affinity TrkA and the low-affinity p75NTR, thereby activating intracellular signaling pathways such as the PI3K/Akt and MAPK/ERK cascades [32]. These pathways inhibit apoptosis and promote neuronal repair and regeneration.

Experimental studies in HIE models have revealed a dynamic time-dependent expression of NGF following hypoxic–ischemic insult. An initial decrease in the acute phase is followed by a compensatory increase in the subacute phase, suggesting an intrinsic mechanism to counteract injury and promote neuroplasticity [33,34]. In addition, the exogenous administration of NGF or NGF mimetics has shown promising results in animal models, including reduced infarct size, preserved neuronal integrity, enhanced neurogenesis, and improved functional outcomes, suggesting significant therapeutic potential [28].

At the earliest phases of this scientific process, Holtzman et al. [28] investigated NGF effects in a neonatal brain injury model. Rat pups subjected to hypoxia–ischemia showed significant brain damage, which was markedly reduced in those treated with NGF. This suggests that NGF provides widespread protection to the developing brain following injury, potentially through mechanisms beyond direct TrkA signaling. Zhong et al. [35] tested another neurotrophin in 2009, examining the effects of exogenous insulin-like growth factor 1 (IGF-1) administration after HIE in neonatal rats. They showed that subcutaneous IGF-1 treatment, particularly when started 24–48 h after injury, reduced brain damage, attenuated apoptosis via the activation of the PI3K/Akt pathway, and improved long-term cognitive and behavioral recovery. Yin et al. investigated the influence of mouse nerve growth factor (mNGF) on glial fibrillary acidic protein (GFAP) expression, a marker of astrocyte activation, in neonatal rats with HIE [36]. The study involved 60 seven-day-old rats divided into control, HIE, and mNGF groups. The mNGF group received daily intramuscular injections of mNGF for five days. Immunohistochemical analysis was used to assess GFAP expression in the hippocampus at days 7 and 14 post surgery. The findings indicated that GFAP expression was significantly higher in the ischemic hippocampus of both the mNGF and HIE groups compared with the control group at both time points (*p* < 0.01). In the mNGF group, GFAP-positive cells were primarily located in the dentate gyrus of the ischemic hippocampus [36]. Over time, GFAP expression in the ischemic hippocampus of the mNGF group increased from day 7 to day 14 (*p* < 0.01), whereas it decreased in the HIE group during the same period (*p* < 0.01), although it remained higher than the control group. The authors concluded that mNGF increases GFAP expression in the hippocampus of neonatal rats with HIE.

In 2017 Wei et al. tested the effects of hyperbaric oxygen (HBO) and NGF on the long-term neural behavior of neonatal rats with HIE [37]. To create the HIE model, the researchers ligated the right common carotid artery of seven-day-old Sprague–Dawley rats, followed by exposure to 8% oxygen and 92% nitrogen for two hours [37]. A total of 40 rats were randomly assigned to five groups: a sham-operated control group, an HIE control group, an HBO-treated group, an NGF-treated group, and a combined NGF + HBO-treated group. At 30 days after birth, the learning and memory abilities of the rats were assessed using the Morris water maze. Sensory motor function was evaluated at 42 days after birth through foot error and limb placement experiments. The results showed that the escape latency in the Morris water maze was significantly shorter in the HBO-treated, NGF-treated, and NGF + HBO-treated groups compared with the HIE control group (*p* < 0.01). Additionally, the piercing indexes, a measure of sensory motor function, were significantly higher in the three treated groups compared with the HIE control group (*p* < 0.01) [37].

In 2025 Landucci et al. investigated the neuroprotective potential of CHF6467, a recombinant modified form of human NGF, in experimental models of neonatal HIE [38]. Their research demonstrated that CHF6467 protected neurons from death and reversed neurotransmission impairment in rodent hippocampal slices subjected to oxygen and glucose deprivation, with even greater efficacy when combined with therapeutic hypothermia. In a neonatal rat HIE model, intranasal CHF6467 significantly reduced brain infarct volume whether administered shortly after injury or several hours later, showing effects comparable to hypothermia alone and a synergistic benefit when both treatments were combined [38]. Furthermore, CHF6467, alone or with hypothermia, improved locomotor coordination and memory, reduced neuroinflammatory markers in the brain, and mitigated the increases in plasma of the neurofilament light chain, a marker of neuroaxonal damage.

**Figure 2:**
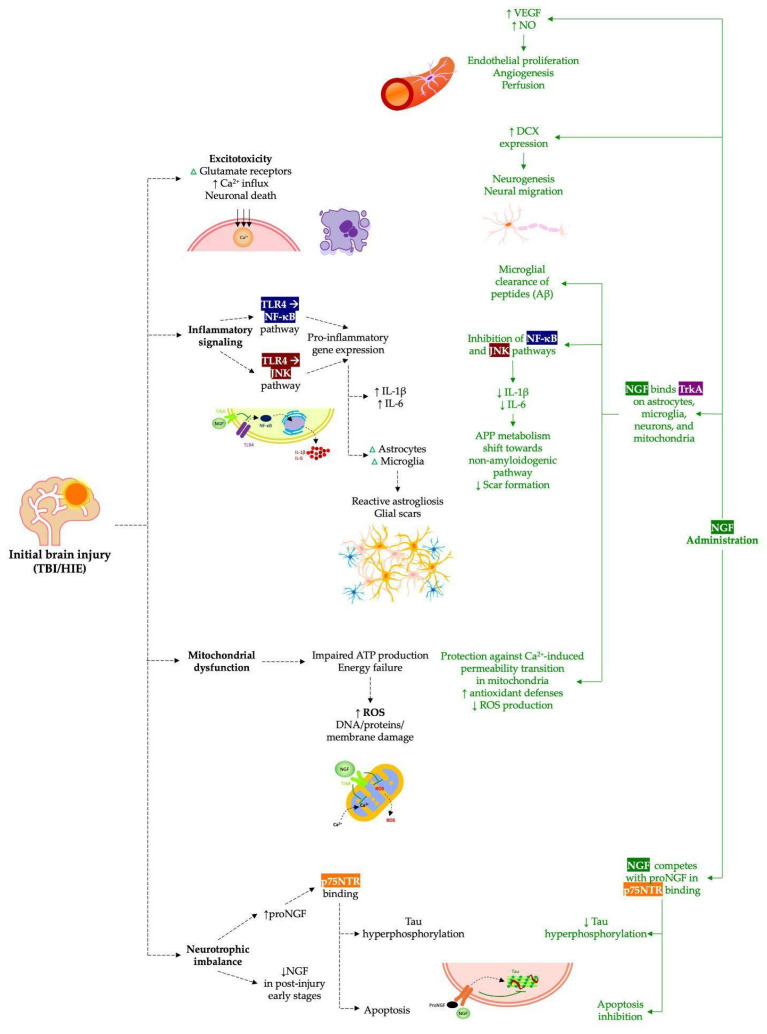
Schematic representation of the molecular and cellular mechanisms involved in initial brain injury (TBI/HIE) and the neuroprotective effects of NGF administration. The pathway on the left (black dotted lines) illustrates the cascade of events following brain injury, including excitotoxicity, inflammatory signaling (via TLR4/NF-κB and TLR4/JNK pathways), mitochondrial dysfunction, neurotrophic imbalance, and subsequent neuronal apoptosis. The pathway on the right (green solid lines) highlights the beneficial actions of NGF, such as promoting angiogenesis, neurogenesis, microglial clearance, the inhibition of inflammatory pathways, mitochondrial protection, and the prevention of tau hyperphosphorylation and apoptosis. Aβ: Amyloid-beta; ATP: Adenosine triphosphate; Ca^2+^: calcium ions; DCX: Doublecortin; DNA: Deoxyribonucleic acid; HIE: Hypoxic–ischemic encephalopathy; IL-1β: Interleukin-1β; IL-6: Interleukin-6; JNK: c-Jun NH 2-terminal kinase; NF-κB: Nuclear Factor-κB; NGF: Nerve growth factor; NO: Nitric oxide; p75NTR: p75 neurotrophin receptor; proNGF: Pro-form of nerve growth factor; ROS: Reactive oxygen species; TBI: Traumatic brain injury; TLR4: Toll-like receptor 4; TrkA: Tropomyosin receptor kinase A; VEGF: Vascular Endothelial Growth Factor.

Although these preclinical results are encouraging, the unique vulnerability of the neonatal brain—stemming from ongoing developmental processes, an immature blood–brain barrier, and a boosted inflammatory response [9]—presents challenges for effective NGF-based interventions. Additionally, the dual nature of NGF signaling through TrkA and p75NTR, which can mediate both survival and apoptotic pathways depending on the cellular context and receptor expression levels, necessitates precise modulation to avoid adverse effects. Figure 2 provides a comprehensive overview of the biomolecular mechanisms by which NGF acts.

At the dawn of clinical translation, Chiaretti et al. [39] showed that two infants, aged 9 and 8 months, with hypoxic–ischemic brain damage secondary to prolonged cardiorespiratory arrest, experienced clinical improvement after intraventricular NGF infusion. Before treatment, both infants were comatose with asymmetrical tetraparesis and GCS scores of 4 and 5, respectively. The intervention involved administering 0.1 mg of purified murine NGF daily for 10 consecutive days via an external drainage catheter into the right cerebral ventricle, starting about 30 days post injury. One month after treatment, their GCS scores improved to 8 and 9, respectively. Follow-up EEG examinations revealed an increased alpha/theta ratio, and MRI showed a reduction in malacic brain areas. A SPECT study in one infant indicated improved regional cerebral perfusion in the right temporal and occipital cortices [39].

A subsequent study by Chiaretti et al. in 2008 investigated the effects of intraventricular NGF infusion in two infants with HIE resulting from cardiorespiratory arrest [40]. By administering 0.1 mg/day of NGF for ten days, starting four months after injury, the researchers observed neurological improvement along with an increased alpha/theta ratio on the EEG and improved cerebral perfusion in the affected areas using SPECT imaging. Remarkably, the study provided the first clinical evidence of NGF stimulating neurogenesis in HIE infants, as indicated by elevated DCX expression in cerebrospinal fluid, suggesting a potential mechanism involving neuroblast migration. While limited by the small sample size and the lack of direct proof linking DCX to functional recovery, this work showed NGF’s dual capacity to improve cerebral blood flow and activate neurogenic pathways even when initiated several months after the initial injury [40].

Furtherly, Chiaretti et al. documented positive outcomes in a cohort of patients who received intraventricular NGF [41]. In this case series four patients—two children with hypoxic–ischemic brain injury, an adult with optic glioma-induced visual loss, and a child with severe crush syndrome—received NGF therapy after failing to recover with standard treatments. Purified NGF from mouse submaxillary glands was administered via an external catheter to the brain, as eye drops, and subcutaneously to the skin. Following NGF treatment, all patients showed notable improvements: neurological and electrophysiological brain function improved in the children with brain injury, visual function improved in the adult with optic glioma, and the ischemic skin lesion gradually healed in the child with crush syndrome. No adverse effects were observed, suggesting that NGF administration may be a safe and effective adjunct therapy for severe hypoxic–ischemic injuries [41].

Subsequently, Fantacci et al. showed that the intraventricular administration of NGF in two children with HIE and prolonged comatose state resulted in significant clinical improvement [34]. Following NGF treatment, both patients exhibited a marked amelioration in EEG patterns and enhanced cerebral perfusion as assessed by SPECT. The observed improvements in motor and cognitive functions were attributed to the neuroprotective effects of NGF on residual viable cholinergic neurons, which may have facilitated the restoration of neuronal networks within the damaged brain [34].

Future research should prioritize elucidating the temporal and spatial patterns of NGF expression in human neonatal brain tissue following HIE, and understanding the interplay between NGF signaling and other molecular pathways involved in neuroinflammation and repair. Comprehensive insights into the role of NGF in neonatal HIE may ultimately contribute to the development of innovative, mechanism-based therapies aimed at mitigating brain injury and promoting long-term neurological recovery in affected infants.

A comprehensive summary is represented in Table 2.

**Table 2:** An overview of studies on NGF in HIE.

Study Type	Population	Intervention	Key Findings	Reference
Preclinical	7 neonatal rats	Intraventricular administration of murine NGF	NGF reduced infarct size (~10% vs. 30–40% controls), enhanced TrkA phosphorylation, induced neuroprotection in cortex and striatum	Holtzman et al., 1996 [28]
Preclinical	60 neonatal rats	Intramuscular injection of murine NGF at a dose of 20 ng/g/day once a day for 5 days	NGF promoted astrocyte activation (increased GFAP expression), supporting neuronal survival and synaptic formation after hypoxic–ischemic brain damage (*p* < 0.01)	Yin et al., 2013 [36]
Preclinical	40 neonatal rats	Intraperitoneal injection of rhNGF at a dose of 0.5 µg administered for 3 days, with/without hyperbaric oxygen	NGF improved learning, memory, and sensory motor function post injury; combined NGF and hyperbaric oxygen had additive benefits (*p* < 0.01)	Wei et al., 2017 [37]
Preclinical	110 neonatal rats	Single intranasal administration of CHF6467 (modified human NGF) at a dose of 20 μg/kg	CHF6467 reduced brain infarct volume, improved neurobehavioral outcomes; synergistic with therapeutic hypothermia (*p* < 0.05)	Landucci et al., 2025 [38]
Clinical	2 infants (8 and 9 months old)	Intraventricular administration of murine NGF at a dose of 0.1 mg/day for 10 days	Improved neurological status (GCS increased from 4/5 to 8/9), enhanced EEG alpha/theta ratio, MRI showed reduction in malacic areas, and SPECT indicated improved cerebral perfusion in affected regions	Chiaretti et al., 2005 [39]
Clinical	2 infants (8 and 13 months old)	Intraventricular administration of murine NGF at a dose of 0.1 mg/day for 10 days	Improved cerebral perfusion, elevated doublecortin expression (neurogenesis marker), enhanced EEG alpha/theta ratio, and neurological recovery	Chiaretti et al., 2008 [40]
Clinical	4 patients: 2 children with hypoxic–ischemic brain damage, an adult patient with an optic glioma-induced visual loss, and a child with a severe crush syndrome of the lower left limb	Administration of murine NGF at a dose of 1 mg via external catheter into brain, eye drops, and subcutaneous skin injection	Amelioration of neurological and electrophysiological function in brain, improvement of visual function, and gradual healing of ischemic skin lesion	Chiaretti et al., 2011 [41]
Clinical	2 children with HIE and coma	Intraventricular administration of murine NGF at a dose of 1 mg, once a day for 10 days	Significant improvement in EEG and SPECT after NGF; motor and cognitive improvement attributed to NGF’s neuroprotective effects on residual cholinergic neurons and network restoration	Fantacci et al., 2013 [34]

## 4. Conclusions and Future Perspectives

The present review underscores the beneficial role of NGF in the contexts of both TBI and HIE. Preclinical investigations have consistently demonstrated the neuroprotective, anti-inflammatory, and regenerative attributes of NGF, resulting in enhanced functional outcomes within animal models of cerebral injury. Nascent clinical evidence related to TBI suggests that the intranasal administration of NGF represents a safe and potentially efficacious strategy for the promotion of neurological recovery, as evidenced by ameliorations in motor skills, cognitive functions, and electrophysiological assessments. Analogously, preliminary clinical inquiries involving HIE infants treated with NGF have proven neurological advancements and indications of neurogenesis. Notwithstanding the current clinical data being constrained by limited sample sizes, the encouraging findings justify further inquiry through more extensive, controlled clinical trials to definitively ascertain the therapeutic efficacy and optimal administration paradigms of NGF for these debilitating neurological conditions. A key finding from the reviewed studies is the absence of reported side effects associated with NGF treatment. However, to comprehensively evaluate the safety profile and therapeutic efficacy of NGF, it is advisable that future research involves larger sample sizes and more rigorous study designs. Such expanded investigations would enhance the statistical power to detect potential adverse effects and better characterize the treatment outcomes. Despite promising preclinical and early clinical data, significant knowledge gaps persist regarding NGF therapy for brain injury. Most clinical evidence is limited to case reports or series, especially in pediatric populations, with a lack of large-scale, randomized controlled trials to robustly assess efficacy and safety. The optimal timing, dosing, and patient selection criteria for NGF administration remain unclear, as does the durability of its therapeutic effects. Mechanistically, while NGF’s roles in neuroprotection, neurogenesis, and the modulation of neuroinflammation are established in animal models, their translation to human pathophysiology is not fully understood. Additionally, long-term safety data are lacking, particularly regarding repeated or high-dose use. Finally, the variability in delivery methods and outcome measures across the studies further complicates the interpretation and generalization of results. Addressing these gaps through rigorous, standardized research is essential to advance NGF therapy toward routine clinical use. Future research endeavors should also prioritize the elucidation of the precise mechanisms underlying NGF’s action within the injured human brain and the identification of potential biomarkers to predict therapeutic responsiveness.

## Data Availability

No new data were created or analyzed in this study. Data sharing is not applicable.

## Abbreviations

The following abbreviations are used in this manuscript:

Aβ	Amyloid-beta
APP	Amyloid precursor protein
ATP	Adenosine triphosphate
CENTRAL	Cochrane Central Register of Controlled Trials
CNS	Central nervous system
CRS-R	Coma Recovery Scale-Revised
CSF	Cerebrospinal fluid
DCX	Doublecortin
DNA	Deoxyribonucleic acid
EEG	Electroencephalography
GCS	Glasgow Coma Scale
GFAP	Glial fibrillary acidic protein
HBO	Hyperbaric oxygen
HIE	Hypoxic–ischemic encephalopathy
hr-NGF	Human-recombinant NGF
IGF-1	Insulin-like growth factor 1
IL-1β	Interleukin-1β
JNK	c-Jun NH 2-terminal kinase
MRI	Magnetic Resonance Imaging
mNGF	Mouse nerve growth factor
NF-κB	Nuclear Factor-κB
NGF	Nerve growth factor
NO	Nitric oxide
p75NTR	p75 neurotrophin receptor
PET/CT	Positron Emission Tomography/Computed Tomography
PRISMA	Preferred Reporting Items for Systematic Reviews and Meta-Analysis
proNGF	Pro-form of nerve growth factor
ROS	Reactive oxygen species
SPECT	Single Photon Emission Computed Tomography
TBI	Traumatic brain injury
TLR4	Toll-like receptor 4
TrkA	Tropomyosin receptor kinase A
VEGF	Vascular Endothelial Growth Factor
VEP	Visual evoked potentials

## References

[1] Lingsma H.F., Roozenbeek B., Steyerberg E.W., Murray G.D., Maas A.I. (2010). Early prognosis in traumatic brain injury: From prophecies to predictions. Lancet Neurol..

[2] Mohamadpour M., Whitney K., Bergold P.J. (2019). The Importance of Therapeutic Time Window in the Treatment of Traumatic Brain Injury. Front. Neurosci..

[3] Ng S.Y., Lee A.Y.W. (2019). Traumatic Brain Injuries: Pathophysiology and Potential Therapeutic Targets. Front. Cell. Neurosci..

[4] Chiaretti A., Barone G., Riccardi R., Antonelli A., Pezzotti P., Genovese O., Tortorolo L., Conti G. (2009). NGF, DCX, and NSE upregulation correlates with severity and outcome of head trauma in children. Neurology.

[5] Kumar A., Loane D.J. (2012). Neuroinflammation after traumatic brain injury: Opportunities for therapeutic intervention. Brain Behav. Immun..

[6] Lin P.H., Kuo L.T., Luh H.T. (2021). The Roles of Neurotrophins in Traumatic Brain Injury. Life.

[7] Ristovska S., Stomnaroska O., Danilovski D. (2022). Hypoxic Ischemic Encephalopathy (HIE) in Term and Preterm Infants. PRILOZI.

[8] Nair J., Kumar V.H.S. (2018). Current and Emerging Therapies in the Management of Hypoxic Ischemic Encephalopathy in Neonates. Children.

[9] Di Sarno L., Curatola A., Cammisa I., Capossela L., Eftimiadi G., Gatto A., Chiaretti A. (2022). Non-pharmacologic approaches to neurological stimulation in patients with severe brain injuries: A systematic review. Eur. Rev. Med. Pharmacol. Sci..

[10] Reichardt L.F. (2006). Neurotrophin-regulated signalling pathways. Philos. Trans. R. Soc. B Biol. Sci..

[11] Vink R., Van Den Heuvel C. (2004). Recent advances in the development of multifactorial therapies for the treatment of traumatic brain injury. Expert Opin. Investig. Drugs.

[12] Allen S.J., Watson J.J., Shoemark D.K., Barua N.U., Patel N.K. (2013). GDNF, NGF and BDNF as therapeutic options for neurodegeneration. Pharmacol. Ther..

[13] Capossela L., Gatto A., Ferretti S., Di Sarno L., Graglia B., Massese M., Soligo M., Chiaretti A. (2024). Multifaceted Roles of Nerve Growth Factor: A Comprehensive Review with a Special Insight into Pediatric Perspectives. Biology.

[14] Gatto A., Capossela L., Conti G., Eftimiadi G., Ferretti S., Manni L., Curatola A., Graglia B., Di Sarno L., Calcagni M.L. (2023). Intranasal human-recombinant NGF administration improves outcome in children with post-traumatic unresponsive wakefulness syndrome. Biol. Direct.

[15] Di Sarno L., Capossela L., Ferretti S., Manni L., Soligo M., Staccioli S., Napoli E., Burattini R., Gatto A., Chiaretti A. (2025). Intranasal Human-Recombinant Nerve Growth Factor Enhances Motor and Cognitive Function Recovery in a Child with Severe Traumatic Brain Injury. Pharmaceuticals.

[16] Page M.J., McKenzie J.E., Bossuyt P.M., Boutron I., Hoffmann T.C., Mulrow C.D., Shamseer L., Tetzlaff J.M., Akl E.A., Brennan S.E. (2021). The PRISMA 2020 statement: An updated guideline for reporting systematic reviews. BMJ.

[17] Chiaretti A., Antonelli A., Mastrangelo A., Pezzotti P., Tortorolo L., Tosi F., Genovese O. (2008). Interleukin-6 and nerve growth factor upregulation correlates with improved outcome in children with severe traumatic brain injury. J. Neurotrauma.

[18] Chiaretti A., Antonelli A., Genovese O., Pezzotti P., Rocco C.D., Viola L., Riccardi R. (2008). Nerve growth factor and doublecortin expression correlates with improved outcome in children with severe traumatic brain injury. J. Trauma.

[19] Chiaretti A., Antonelli A., Riccardi R., Genovese O., Pezzotti P., Di Rocco C., Tortorolo L., Piedimonte G. (2008). Nerve growth factor expression correlates with severity and outcome of traumatic brain injury in children. Eur. J. Paediatr. Neurol..

[20] Manni L., Conti G., Chiaretti A., Soligo M. (2023). Intranasal nerve growth factor for prevention and recovery of the outcomes of traumatic brain injury. Neural Regen. Res..

[21] Chiaretti A., Conti G., Falsini B., Buonsenso D., Crasti M., Manni L., Soligo M., Fantacci C., Genovese O., Calcagni M.L. (2017). Intranasal Nerve Growth Factor administration improves cerebral functions in a child with severe traumatic brain injury: A case report. Brain Inj..

[22] Tian L., Guo R., Yue X., Lv Q., Ye X., Wang Z., Chen Z., Wu B., Xu G., Liu X. (2012). Intranasal administration of nerve growth factor ameliorate β-amyloid deposition after traumatic brain injury in rats. Brain Res..

[23] Lv Q., Fan X., Xu G., Liu Q., Tian L., Cai X., Sun W., Wang X., Cai Q., Bao Y. (2013). Intranasal delivery of nerve growth factor attenuates aquaporins-4-induced edema following traumatic brain injury in rats. Brain Res..

[24] Lv Q., Lan W., Sun W., Ye R., Fan X., Ma M., Yin Q., Jiang Y., Xu G., Dai J. (2014). Intranasal nerve growth factor attenuates tau phosphorylation in brain after traumatic brain injury in rats. J. Neurol. Sci..

[25] Manni L., Leotta E., Mollica I., Serafino A., Pignataro A., Salvatori I., Conti G., Chiaretti A., Soligo M. (2023). Acute Intranasal Treatment with Nerve Growth Factor Limits the Onset of Traumatic Brain Injury in Young Rats. Br. J. Pharmacol..

[26] Young J., Pionk T., Hiatt I., Geeck K., Smith J.S. (2015). Environmental enrichment aides in functional recovery following unilateral controlled cortical impact of the forelimb sensorimotor area however intranasal administration of nerve growth factor does not. Brain Res. Bull..

[27] Capossela L., Graglia B., Ferretti S., Di Sarno L., Gatto A., Calcagni M.L., Di Giuda D., Cocciolillo F., Romeo D.M., Manni L. (2024). Intranasal human-recombinant nerve growth factor administration improves cognitive functions in a child with severe traumatic brain injury. Eur. Rev. Med. Pharmacol. Sci..

[28] Holtzman D.M., Sheldon R.A., Jaffe W., Cheng Y., Ferriero D.M. (1996). Nerve growth factor protects the neonatal brain against hypoxic-ischemic injury. Ann. Neurol..

[29] Gillam-Krakauer M., Shah M., Gowen C.W. (2025). Birth Asphyxia.

[30] Gunn A.J., Thoresen M. (2019). Neonatal encephalopathy and hypoxic-ischemic encephalopathy. Handb. Clin. Neurol..

[31] Ranjan A.K., Gulati A. (2023). Advances in Therapies to Treat Neonatal Hypoxic-Ischemic Encephalopathy. J. Clin. Med..

[32] Conroy J.N., Coulson E.J. (2022). High-affinity TrkA and p75 neurotrophin receptor complexes: A twisted affair. J. Biol. Chem..

[33] Lee T.H., Kato H., Chen S.T., Kogure K., Itoyama Y. (1998). Expression of nerve growth factor and trkA after transient focal cerebral ischemia in rats. Stroke.

[34] Fantacci C., Capozzi D., Ferrara P., Chiaretti A. (2013). Neuroprotective role of nerve growth factor in hypoxic-ischemic brain injury. Brain Sci..

[35] Zhong J., Zhao L., Du Y., Wei G., Yao W.G., Lee W.H. (2009). Delayed IGF-1 treatment reduced long-term hypoxia-ischemia-induced brain damage and improved behavior recovery of immature rats. Neurol. Res..

[36] Yin X., Dong L., Wei W., Wang Y., Chai Y., Feng Z. (2013). Effect of mouse nerve growth factor on the expression of glial fibrillary acidic protein in hippocampus of neonatal rats with hypoxic-ischemic brain damage. Exp. Ther. Med..

[37] Wei L., Ren Q., Zhang Y., Wang J. (2017). Effects of hyperbaric oxygen and nerve growth factor on the long-term neural behavior of neonatal rats with hypoxic ischemic brain damage. Acta Cir. Bras..

[38] Landucci E., Mango D., Carloni S., Mazzantini C., Pellegrini-Giampietro D.E., Saidi A., Balduini W., Schiavi E., Tigli L., Pioselli B. (2025). Beneficial effects of CHF6467, a modified human nerve growth factor, in experimental neonatal hypoxic-ischaemic encephalopathy. Br. J. Pharmacol..

[39] Chiaretti A., Genovese O., Riccardi R., Di Rocco C., Di Giuda D., Mariotti P., Pulitanò S., Piastra M., Polidori G., Colafati G.S. (2005). Intraventricular nerve growth factor infusion: A possible treatment for neurological deficits following hypoxic-ischemic brain injury in infants. Neurol. Res..

[40] Chiaretti A., Antonelli A., Genovese O., Fernandez E., Giuda D., Mariotti P., Riccardi R. (2008). Intraventricular nerve growth factor infusion improves cerebral blood flow and stimulates doublecortin expression in two infants with hypoxic-ischemic brain injury. Neurol. Res..

[41] Chiaretti A., Falsini B., Aloe L., Pierri F., Fantacci C., Riccardi R. (2011). Neuroprotective role of nerve growth factor in hypoxicischemic injury. From brain to skin. Arch. Ital. Biol..

